# Failed mitochondrial import and impaired proteostasis trigger SUMOylation of mitochondrial proteins

**DOI:** 10.1074/jbc.M117.817833

**Published:** 2017-11-28

**Authors:** Florian Paasch, Fabian den Brave, Ivan Psakhye, Boris Pfander, Stefan Jentsch

**Affiliations:** From the ‡ Department of Molecular Cell Biology and; the § Research Group DNA Replication and Genome Integrity, Max Planck Institute of Biochemistry, Am Klopferspitz 18, 82152 Martinsried, Germany

**Keywords:** 70-kilodalton heat shock protein (HSP70), mitochondria, proteasome, proteostasis, small ubiquitin-like modifier (SUMO), mitochondrial proteins, protein quality control, SUMOylation

## Abstract

Modification by the ubiquitin-like protein SUMO affects hundreds of cellular substrate proteins and regulates a wide variety of physiological processes. While the SUMO system appears to predominantly target nuclear proteins and, to a lesser extent, cytosolic proteins, hardly anything is known about the SUMOylation of proteins targeted to membrane-enclosed organelles. Here, we identify a large set of structurally and functionally unrelated mitochondrial proteins as substrates of the SUMO pathway in yeast. We show that SUMO modification of mitochondrial proteins does not rely on mitochondrial targeting and, in fact, is strongly enhanced upon import failure, consistent with the modification occurring in the cytosol. Moreover, SUMOylated forms of mitochondrial proteins particularly accumulate in HSP70- and proteasome-deficient cells, suggesting that SUMOylation participates in cellular protein quality control. We therefore propose that SUMO serves as a mark for nonfunctional mitochondrial proteins, which only sporadically arise in unstressed cells but strongly accumulate upon defective mitochondrial import and impaired proteostasis. Overall, our findings provide support for a role of SUMO in the cytosolic response to aberrant proteins.

## Introduction

Posttranslational modification by the small ubiquitin-like modifier (SUMO)^4^ is of fundamental importance for the regulation of a wide variety of physiological processes. Consistent with the large number of cellular SUMO substrates and its crucial roles in cell homeostasis, SUMOylation is essential for viability in most eukaryotes. Moreover, SUMO has been widely implicated in cellular responses to stress, including hypoxic, osmotic, genotoxic, and nutrient stress (1). In particular, SUMOylation is strongly induced under conditions of proteotoxic stress and targets a diverse array of substrate proteins upon protein misfolding caused by heat shock (2–4) or proteasome inhibition (5–7).

Most SUMO substrates identified to date are nuclear proteins (8) and also most SUMO conjugating and deconjugating enzymes show a primarily nuclear localization (9–12). However, SUMOylation is not restricted to the nucleus and several cytosolic SUMO targets have been identified as well (13). Well-studied examples of SUMO substrates outside the nucleus are the septins in budding yeast, which become SUMOylated by the cytosolic pool of the SUMO E3 ligase Siz1 during mitosis (14–19) and deSUMOylated by the SUMO-specific isopeptidase Ulp1 during cytokinesis (14, 15, 20).

Interestingly, SUMO substrates in the cytosol also include proteins that are located at the interfaces of the plasma membrane and cellular organelles such as the nucleus, the endoplasmic reticulum, and mitochondria (13). This group of substrates includes the GTPase DRP1 in mammals, which currently is the only well-characterized SUMO substrate that localizes to mitochondria. DRP1 associates with the cytosolic side of the outer mitochondrial membrane. SUMOylation of DRP1 was found to be mediated by the mitochondrial anchored SUMO E3 ligase MAPL (21) and to promote mitochondrial fission under normal growth conditions (22) as well as during apoptosis (23).

Ubiquitin has also been identified as regulator of mitochondrial homeostasis and has been linked to mitochondrial protein quality control (24, 25). Notably, the ubiquitin-proteasome system was shown to mediate the degradation of nonimported mitochondrial proteins under physiological conditions (26) and acutely upon import failure (26–30). In this scenario, ubiquitin is conjugated to proteins that normally localize to and function within the inner mitochondrial subcompartments (26, 27). By contrast, SUMO modification of proteins from inner mitochondrial subcompartments has not been analyzed to date, even though previous large-scale “SUMOylome” studies have suggested a small number of putative SUMO substrates from these compartments (31–36).

Starting from a mass spectrometry (MS) approach, we provide here evidence that a substantial fraction of the mitochondrial proteome is targeted by the SUMO modification system. We corroborate our MS data by individually confirming the SUMO modification of several mitochondrial matrix proteins *in vivo*. In agreement with the presence of SUMO enzymes in the cytosol but not in the mitochondrial matrix, we find that the SUMOylation of mitochondrial proteins does not rely on mitochondrial import. By contrast, our data rather indicate that SUMOylation of mitochondrial proteins is strongly enhanced upon import failure. Moreover, whereas SUMO modification of these substrates occurs only sporadically in unstressed cells, it is particularly induced when canonical components of the proteostasis network, such as the HSP70 system or the proteasome, are defective. Finally, we propose a model in which the SUMO modification pathway targets nonfunctional mitochondrial proteins as an element of cellular protein quality control.

## Results

### Mitochondrial proteins are modified by SUMO in vivo

Following our long-standing interest in the SUMO system, we screened for novel SUMO substrates using a strategy that involves the purification of His-tagged SUMO (^His^SUMO) conjugates from yeast cells and analysis of the enriched proteins using quantitative mass spectrometry (37, 38). Previous large-scale studies in budding yeast had suggested a small number of mitochondrial proteins as potential SUMO substrates (31–36). Notably, we consistently identified peptides of several different mitochondrial proteins in our ^His^SUMO purifications. By compiling the results of multiple MS experiments, our approach revealed a set of 89 inner mitochondrial proteins as potential SUMO substrates (Table S1). For 61 of these proteins we also identified a total of 81 SUMOylation sites, further suggesting that these proteins are modified *in vivo* (Table S1).

**Figure 1.**
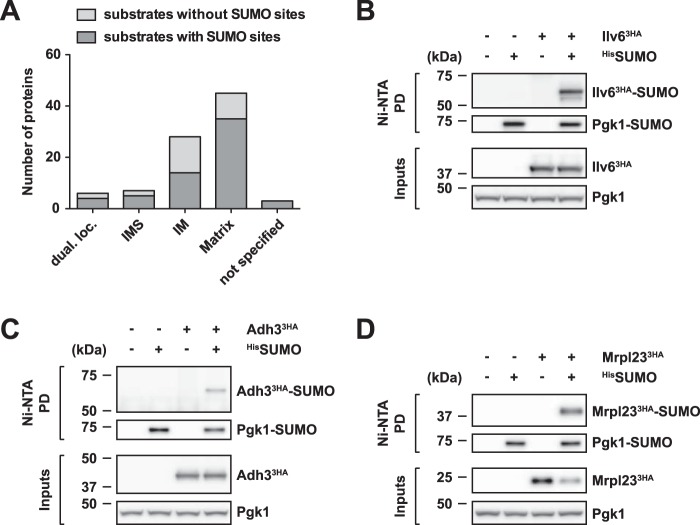
**Mitochondrial proteins are modified by SUMO *in vivo*.** *A*, submitochondrial distribution of 89 potential SUMO substrates, which were identified by a mass spectrometry–based approach. Putative SUMO substrates identified with SUMO acceptor site(s) are indicated in *dark gray. dual loc*., dual localization; *IMS*, intermembrane space; and *IM*, inner membrane. *B–D*, mitochondrial matrix proteins Ilv6, Adh3, and Mrpl23 are SUMO substrates. Shown are ^His^SUMO Ni-NTA pulldowns from wild-type cells and cells expressing epitope-tagged proteins as indicated. His-tagged SUMO (^His^SUMO) was expressed from the *ADH1* promoter and C-terminally 3HA-tagged Ilv6 (*B*), 3HA-tagged Adh3 (*C*), or 3HA-tagged Mrpl23 (*D*) from the endogenous promoter (*B* and *C*) or *ADH1* promoter (*D*). Proteins were detected by Western blotting using HA epitope- and Pgk1-specific antibodies. Pgk1 SUMOylation was analyzed to control for pulldown efficiency, and unmodified Pgk1 served as control.

Among the 89 potential mitochondrial SUMO substrates, we found components of all inner mitochondrial subcompartments (intermembrane space, inner membrane, and matrix) (Fig. 1*A*). A comparison with the localization of known mitochondrial proteins listed in the Yeast Deletion and Proteomics of Mitochondria (YDPM) database (39) suggested that the number of SUMO substrates from each subcompartment largely scaled with the overall number of proteins in each subcompartment. Seemingly, therefore, submitochondrial localization is not a determinant for SUMO modification. Importantly, only a small fraction of substrates (six proteins) were annotated as having a dual localization (mitochondrial and cytosolic). We therefore conclude that a large number of proteins that function in mitochondria are modified by SUMO *in vivo*.

To ascertain the MS results, we analyzed the SUMOylation of several structurally and functionally unrelated mitochondrial proteins individually. Using denaturing Ni-NTA pulldowns and subsequent Western blot analysis we were able to confirm that these mitochondrial matrix proteins are indeed modified by SUMO. These confirmed SUMO substrates include Ilv6 (Fig. 1*B*), a protein involved in branched-chain amino acid biosynthesis (40, 41), Adh3 (Fig. 1*C*), a mitochondrial alcohol dehydrogenase isoform (42, 43), and Mrpl23 (Fig. 1*D*), a mitochondrial ribosomal protein (44).

We next aimed to identify the SUMO acceptor sites on mitochondrial SUMO substrates. To this end, we systematically replaced individual lysine residues on Ilv6, Adh3, and Mrpl23 to arginine. For Ilv6, this identified lysine 260 as major SUMO acceptor site (Fig. S1, *A* and *B*), but additional removal of three adjacent lysine residues (Lys-218, Lys-284, and Lys-296) in a stepwise manner further reduced SUMOylation and a mutant variant lacking all four lysine residues (Ilv6^3HA^-K218R, K260R, K284R, K296R termed Ilv6^3HA^-4KR) did not show any detectable SUMOylation (Fig. S1, *A* and *B*). For Adh3, we found lysine 305 as major SUMO acceptor site (Fig. S1, *C* and *D*) and for Mrpl23, replacement of the two most C-terminal lysine residues by arginine reduced the levels of SUMO conjugates to less than 50% (Fig. S1, *E* and *F*).

### SUMOylation of mitochondrial proteins requires the SUMO E3 ligases Siz1 and Siz2

**Figure 2.**
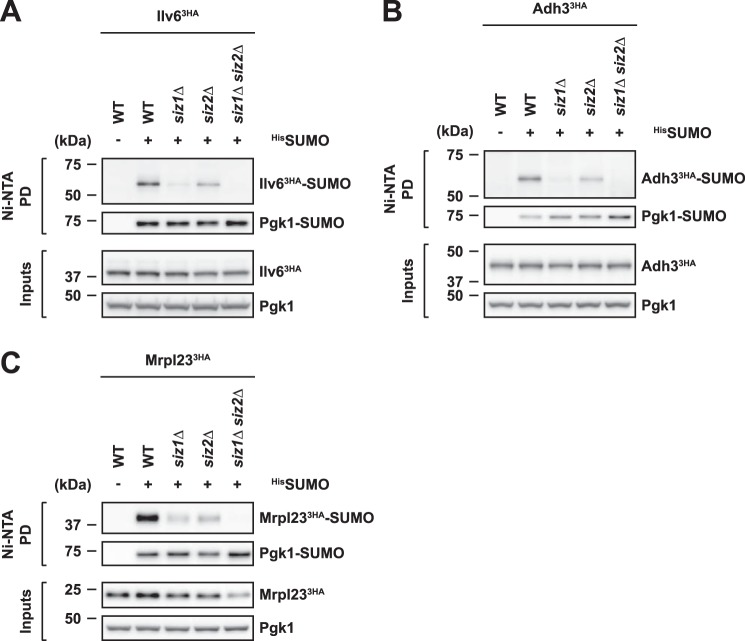
**SUMO modification of mitochondrial proteins requires SUMO E3 ligases Siz1 and Siz2.** *A–C*, SUMOylation of Ilv6, Adh3, and Mrpl23 depends on Siz1 and to a lesser extent on Siz2. Denaturing ^His^SUMO Ni-NTA pulldowns from wild-type (*WT*) cells and cells lacking Siz1 (*siz1*Δ), Siz2 (*siz2*Δ), or both (*siz1*Δ *siz2*Δ). Strains express C-terminally 3HA-tagged Ilv6 (*A*), 3HA-tagged Adh3 (*B*), or 3HA-tagged Mrpl23 (*C*).

We next asked whether SUMOylation of mitochondrial proteins requires specific SUMO E3 ligases. Analysis of Ilv6 SUMOylation in cells lacking the known SUMO E3 ligases Siz1 (*siz1*Δ) or Siz2 (*siz2*Δ) indicated that the SUMO modification of Ilv6 is catalyzed by Siz1 and to a minor extent by Siz2 (Fig. 2*A*). Accordingly, Ilv6 SUMOylation was undetectable by Western blotting in samples from cells lacking both Siz1 and Siz2 (*siz1*Δ *siz2*Δ) (Fig. 2*A*). Moreover, we found strikingly similar roles for Siz1 and Siz2 in the SUMO modification of Adh3 (Fig. 2*B*) and Mrpl23 (Fig. 2*C*). Thus, all tested SUMO substrates require the same SUMO E3 ligases of the conserved Siz/PIAS (protein inhibitor of activated STAT) family for modification.

### SUMOylation of mitochondrial proteins is enhanced upon import failure

**Figure 3.**
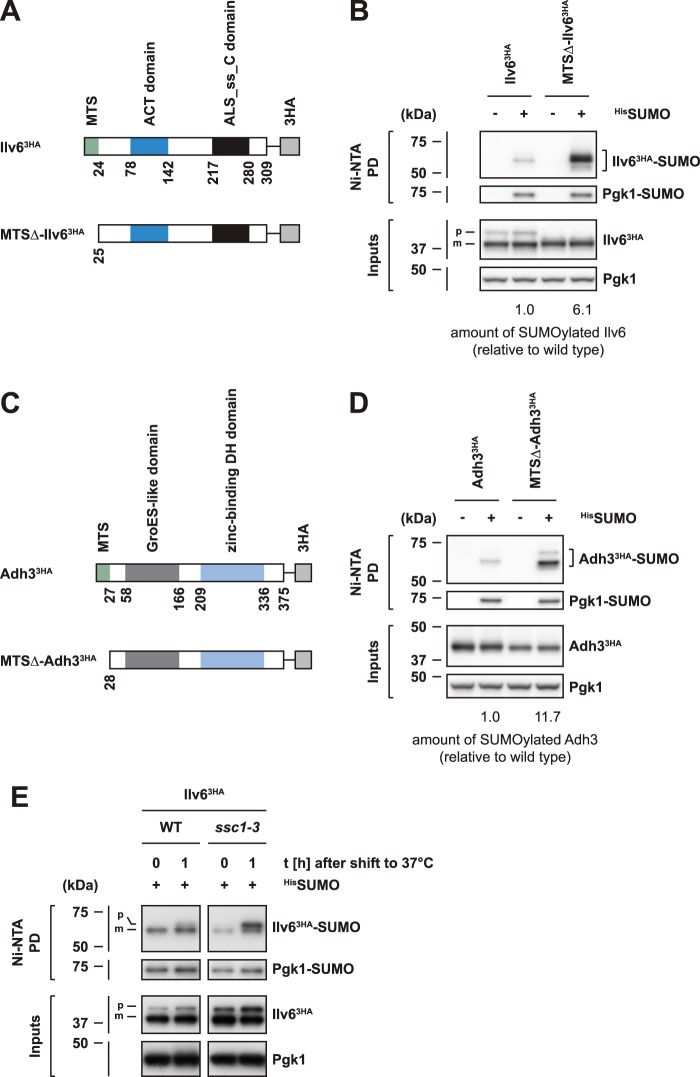
**SUMO modification of mitochondrial proteins is enhanced upon import failure.** *A–D*, SUMOylation of Ilv6 and Adh3 is enhanced in the absence of a mitochondrial targeting signal. *A* and *C*, schematic representation of Ilv6^3HA^ (*A*) or Adh3^3HA^ (*C*) and corresponding mutant variants lacking the matrix-targeting sequence (*MTS*). *ACT domain*, named after aspartate kinase, chorismate mutase, TyrA; *ALS*_*ss*_*C domain*, acetolactate synthase, small subunit, C-terminal domain; *zinc-binding DH domain*, zinc-binding dehydrogenase domain. *B* and *D*, denaturing ^His^SUMO Ni-NTA pulldowns from cells harboring plasmids that express wild-type Ilv6 (*B*) or Adh3 (*D*) and corresponding import-incompetent mutant variants as indicated from the *GAL1* promoter. Data information: The ratios of SUMOylated/unmodified proteins (*B* and *D*) were determined by Western blot quantification using ImageJ and normalized to the wild-type proteins. *E*, a SUMOylated Ilv6 precursor accumulates upon inactivation of mitochondrial HSP70 (Ssc1). Denaturing ^His^SUMO Ni-NTA pulldowns from wild-type (*WT*) and temperature-sensitive *ssc1-3* cells expressing C-terminally 3HA-tagged Ilv6 from the endogenous promoter. Cells were grown at 25 °C and shifted to 37 °C for 1 h. *Bands* corresponding to the unmodified or monoSUMOylated precursor protein (*p*) or mature form (*m*) are labeled.

The vast majority of mitochondrial proteins are synthesized on cytosolic ribosomes and subsequently imported into mitochondria (45, 46). We therefore asked whether SUMOylation of mitochondrial proteins is linked to the import process or requires the presence of a mitochondrial targeting signal. Classical mitochondrial targeting signals are N-terminal presequences, which in most cases are proteolytically removed from mitochondrial precursor proteins during import. Presequences frequently target proteins into the mitochondrial matrix and therefore are also referred to as matrix-targeting sequences (MTS) (45). Accordingly, we generated an Ilv6 mutant variant (MTSΔ-Ilv6^3HA^), which lacks the N-terminal MTS (amino acids 1–24) required for mitochondrial import (Fig. 3*A*). Microscopic analysis of corresponding GFP fusion proteins demonstrated that removal of the 24 N-terminal amino acids of Ilv6 is indeed sufficient to prevent mitochondrial import, thereby causing a presumably cytosolic localization of the mutant protein variant (Fig. S2, *A* and *B*). Importantly, this mutant was efficiently SUMOylated (Fig. 3*B*) and the modification was again dependent on the SUMO E3 ligases Siz1 and Siz2 (Fig. S2*C*) and occurred at a similar set of SUMO acceptor sites as for wild-type Ilv6 (Fig. S2*D*). Therefore, SUMO modification of Ilv6 does not rely on mitochondrial import and does not require the presence of an MTS. In fact, MTS deletion even strongly enhanced the SUMOylation of Ilv6 (Fig. 3*B*), indicating that SUMO modification is induced by import failure.

We also analyzed an import-incompetent variant of Adh3 (MTSΔ-Adh3^3HA^) (Fig. 3*C*). Again, SUMO modification of the import-incompetent variant was enhanced compared with the wild-type protein (Fig. 3*D*). Furthermore, SUMOylation of import-incompetent Adh3 also required the SUMO E3 ligases Siz1 and Siz2 (Fig. S2*E*) and the predominantly targeted lysine 305 (Fig. S2*F*), similar to wild-type Adh3. We therefore conclude that import-incompetent mutant variants of mitochondrial proteins are SUMOylated with requirements as wild-type substrates, but that deletion of targeting sequences strongly enhances the modification. This may suggest that SUMOylation of wild-type proteins specifically occurs upon sporadic mistargeting in unstressed cells and under conditions where mitochondrial protein import is impaired. Indeed, we observed an accumulation of SUMOylated Ilv6 precursors (p) in strains defective in mitochondrial import (Fig. 3*E*), which harbor a temperature-sensitive mutant variant of mitochondrial HSP70 (*ssc1-3*) (47). Furthermore, we specifically observed the accumulation of SUMOylated Ilv6 precursors in the *ssc1-3* mutant, whereas in wild-type cells Ilv6-SUMO conjugates appeared to be proteolytically processed (Fig. 3*E*; note the shift of the precursor (p) compared with the mature form (m)). This indicates that the major pool of Ilv6-SUMO conjugates in unstressed wild-type cells possesses a proteolytically processed N terminus. It is therefore conceivable that these protein species have at some point initiated mitochondrial import, but that they become modified by SUMO in the cytosol.

### SUMOylation of mitochondrial proteins is regulated by the SSA family of HSP70 chaperones

**Figure 4.**
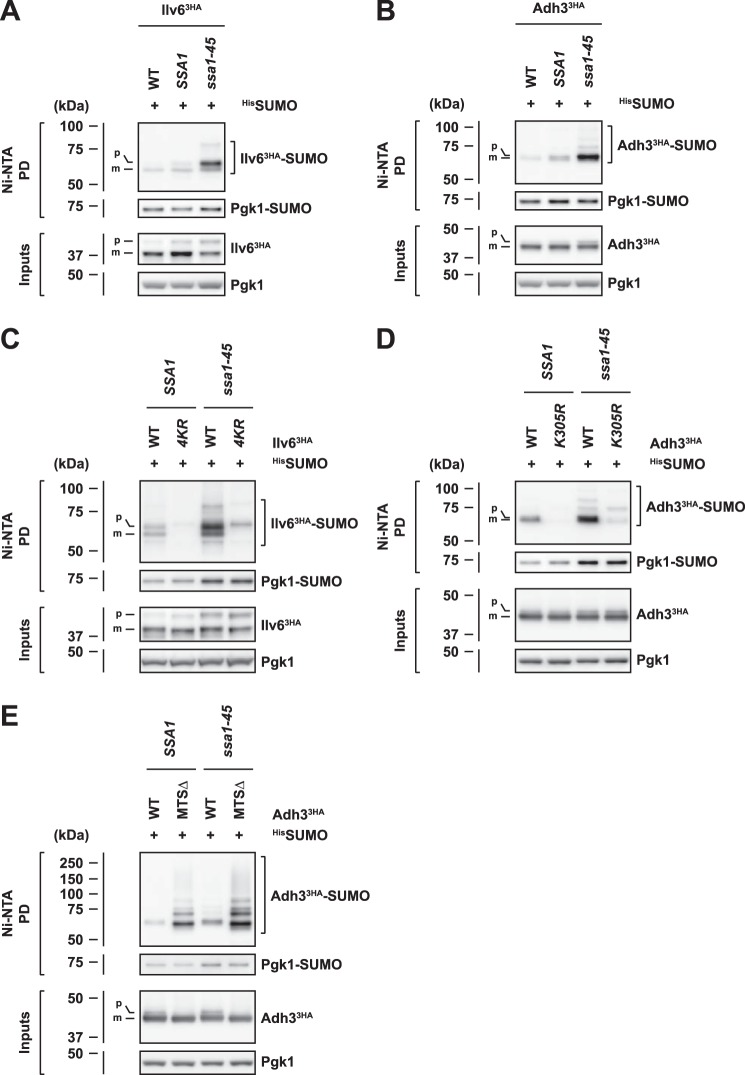
**SUMO modification of mitochondrial proteins is enhanced in *SSA* family HSP70 mutant cells.** *A* and *B*, inactivation of Ssa1–4 causes accumulation of Ilv6 (*A*) and Adh3 (*B*) SUMO conjugates and of the corresponding precursors. Denaturing ^His^SUMO Ni-NTA pulldowns from wild-type (*WT*), *SSA1* (*SSA1 ssa2*Δ *ssa3*Δ *ssa4*Δ) and *ssa1-45* (*ssa1-45 ssa2*Δ *ssa3*Δ *ssa4*Δ) cells expressing 3HA-tagged Ilv6 (*A*) or 3HA-tagged Adh3 (*B*) from the endogenous promoter. *Bands* corresponding to the unmodified or monoSUMOylated precursor proteins (*p*) or mature forms (*m*) are labeled. *C* and *D*, SUMO modification after *SSA* impairment occurs via specific and additionally accessible modification sites. Denaturing ^His^SUMO Ni-NTA pulldowns from *SSA1* and *ssa1-45* cells expressing 3HA-tagged wild-type (*WT*) Ilv6 (*C*) or 3HA-tagged wild-type Adh3 (*D*) and the indicated lysine to arginine mutants from the *ADH1* (*C*) or *TDH3* (*D*) promoter. *E*, *SSA* impairment and deletion of the Adh3 MTS cause an additive enhancement in Adh3 SUMOylation. Denaturing ^His^SUMO Ni-NTA pulldowns from *SSA1* and *ssa1-45* cells harboring plasmids that express wild-type (*WT*) or MTS-lacking (MTSΔ) 3HA-tagged Adh3 from the *TDH3* promoter. *Bands* corresponding to the Adh3 precursor (*p*) or mature form (*m*) are labeled.

Based on our observation that the SUMOylation of mitochondrial proteins can occur in the cytosol, we speculated that the modification might be affected by factors which bind nonimported mitochondrial precursor proteins. Several factors are involved in posttranslational protein import into mitochondria (48–50), of which the *SSA* family HSP70 proteins (Ssa1–4) are of particular importance in budding yeast (51, 52). We therefore used cells in which HSP70 function was diminished by deletion of three out of four *SSA* genes (*ssa2*Δ *ssa3*Δ *ssa4*Δ) and additional expression of either wild-type *SSA1* or the hypomorphic mutant variant *ssa1-45* (53). HSP70 chaperones have been shown to bind to mitochondrial precursor proteins, to maintain them in an import-competent state, and to prevent their aggregation (52, 54, 55). Consistently, we observed an increased aggregation propensity of the Ilv6 precursor (p) compared with the processed mitochondrial form (m) of the protein (Fig. S3*A*). Moreover, the levels of the Ilv6 precursor were mildly increased when HSP70 activity was compromised (Fig. S3*A*). This indicates that *SSA* family chaperones are indeed involved in the mitochondrial import of Ilv6. Strikingly, we also detected a strong accumulation of SUMOylated Ilv6 precursors in *SSA* mutants, particularly in *ssa1-45* cells (Fig. 4*A*), which was suppressed by ectopic expression of Ssa1 from the strong constitutive *ADH1* promoter (Fig. S3, *B* and *C*). Notably, in addition to monoSUMOylated Ilv6, we detected further bands with a slower migration behavior in *ssa1-45* cells, suggesting the presence of Ilv6 species modified with multiple SUMO moieties (Fig. 4*A*). Likewise, the levels of SUMOylated Adh3 were elevated in the *ssa1-45* background (Fig. 4*B*), indicating that a functionally compromised *SSA* chaperone system generally causes enhanced SUMO modification of mitochondrial proteins. Interestingly, when we further characterized Ilv6 and Adh3 SUMOylation in *SSA* mutants, we additionally noticed the modification of lysine residues, which were not detectable by Western blotting in wild-type cells (Fig. 4, *C* and *D* and Fig. S3*D*).

To test if the enhanced SUMOylation of mitochondrial proteins in *SSA* mutant cells would simply result from an import defect, we introduced the import-incompetent variant of Adh3 (MTSΔ-Adh3^3HA^) in *SSA1* and *ssa1-45* cells (Fig. 4*E*). Strikingly, however, we observed a further enhancement of Adh3 SUMOylation, suggesting that import failure and *SSA* deficiency exert an additive effect on the SUMOylation of mitochondrial proteins. We thus conclude that defective mitochondrial import and functional impairment of the *SSA* HSP70 system are additive triggers for SUMOylation of mitochondrial proteins.

### Enhanced SUMOylation of mitochondrial proteins in proteasome mutants

Molecular chaperones are central hubs of cellular protein quality control. In addition to their function in protein folding, they also act as decision makers and target terminally misfolded proteins for degradation by the ubiquitin-proteasome system (56, 57). We therefore asked whether the SUMO modification of mitochondrial proteins would be influenced by the cells' ability to degrade proteins via the proteasome. To abrogate proteasome function, we took advantage of the *cim3-1* temperature-sensitive mutant, which induces a defect in the 19S regulatory proteasome subunit Rpt6 (58), or used the proteasome inhibitor MG132.

**Figure 5.**
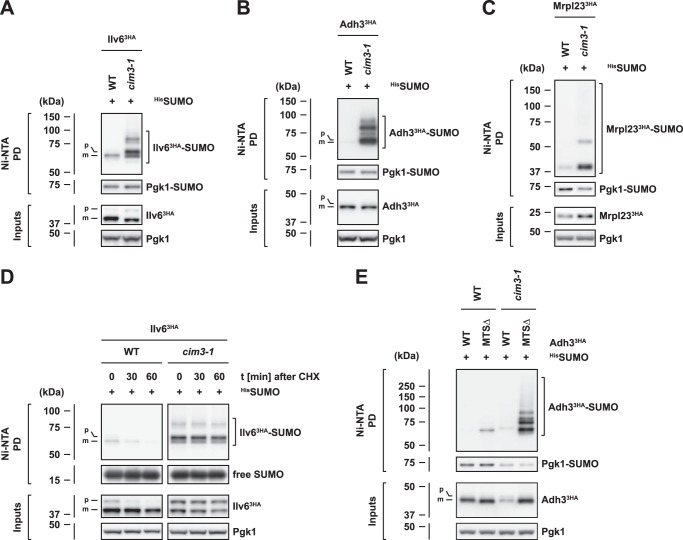
**Accumulation of SUMO-modified mitochondrial proteins in proteasome mutants.** *A–C*, SUMO-modified forms of mitochondrial proteins accumulate under conditions of proteasome impairment. Denaturing ^His^SUMO Ni-NTA pulldowns from wild-type (*WT*) and *cim3-1* cells. The strains express 3HA-tagged Ilv6 (*A*) or 3HA-tagged Adh3 (*B*) from their endogenous promoters or harbor plasmids that express 3HA-tagged Mrpl23 (*C*) from the *ADH1* promoter. *Bands* corresponding to the unmodified or monoSUMOylated precursor proteins (*p*) or mature forms (*m*) are labeled. *D*, Ilv6 precursors and SUMOylated Ilv6 species are stabilized in the *cim3-1* mutant. Expression shut-off assay of Ilv6-SUMO conjugates. Yeast cells were treated with 0.5 mg/ml cycloheximide (*CHX*) and samples were harvested at the indicated time points. Denaturing Ni-NTA pulldowns were performed to isolate ^His^SUMO conjugates from cells expressing 3HA-tagged Ilv6 from the endogenous promoter. *Bands* corresponding to the unmodified or monoSUMOylated precursor protein (*p*) or mature form (*m*) are labeled. *E*, proteasome impairment and deletion of the Adh3 MTS cause an additive enhancement in Adh3 SUMOylation. Denaturing ^His^SUMO Ni-NTA pulldowns from wild-type (*WT*) and *cim3-1* cells harboring plasmids that express Adh3 variants as indicated from the *ADH1* promoter.

Strikingly, when we analyzed SUMO conjugates in *cim3-1* cells, we found an accumulation of multiple SUMOylated species of Ilv6 (Fig. 5*A*), Adh3 (Fig. 5*B*), and Mrpl23 (Fig. 5*C*), indicating that proteasomal degradation strongly impacts the SUMOylation of mitochondrial proteins. Moreover, as judged from the SUMOylation patterns of Adh3 and Ilv6 in *cim3-1* cells, which were similar to those observed in *SSA* mutants, proteasome inhibition also led to an accumulation of SUMOylated precursor proteins. This idea was further confirmed by analysis of Ilv6 SUMOylation in cells treated with the proteasome inhibitor MG132 (Fig. S4A). Notably, we consistently detected a slight accumulation of precursor proteins of Ilv6 and Adh3 in *cim3-1* and MG132-treated cells (Fig. 5, *A* and *B* and Fig. S4*A*), which is in line with previous studies that have implicated the ubiquitin-proteasome system in the clearance of nonimported mitochondrial proteins from the cytosol (26–30). Taken together, these observations suggest that SUMOylation targets nonimported mitochondrial precursor proteins, which fail to be degraded in response to proteasome inhibition.

In addition to our observation of increased mitochondrial protein SUMOylation upon proteasome inhibition, we also tested whether the SUMO conjugates themselves would be substrates of the proteasome and therefore stabilized in proteasome mutant cells. Therefore, we combined a translation shut-off experiment (cycloheximide shut-off) with denaturing Ni-NTA pulldowns to monitor SUMOylated Ilv6 over time. First, we observed that the unmodified Ilv6 precursor is rapidly degraded in wild-type cells, but strongly stabilized in the *cim3-1* mutant (Fig. 5*D*; see *inputs*). This indicates that Ilv6 precursors, which fail to be imported into mitochondria, are degraded in a proteasome-dependent manner. Second, we observed that SUMOylated Ilv6 is rapidly turned over in wild-type cells and almost completely stabilized in *cim3-1* cells (Fig. 5*D*; see *Ni-NTA pulldowns*). Overall, SUMOylated forms of Ilv6 are turned over by the proteasome in remarkably similar fashion as the Ilv6 precursor.

Consistent with the proteasomal clearance of nonimported Ilv6 species, we found that the import-incompetent Ilv6 mutant variant, which lacks its MTS (MTSΔ-Ilv6^3HA^), is degraded in a proteasome-dependent manner (Fig. S4*B*). However, removal of the four major SUMOylation sites or even completely abolishing SUMO modification by deletion of the SUMO E3 ligases Siz1 and Siz2 did not noticeably delay the degradation of import-incompetent Ilv6 (Fig. S4, *C* and *D*). Therefore, although we cannot exclude that a minor pool of nonimported Ilv6 is targeted for degradation in a SUMO-dependent manner, this indicates that SUMOylation is not used as a widespread signal for proteasomal clearance of the majority of nonimported mitochondrial proteins. We conclude that SUMO rather serves as a mark for nonimported mitochondrial proteins, particularly in the absence of proteasomal degradation.

Given our observation that SUMOylation of mitochondrial proteins is also enhanced upon import failure, we predicted that SUMOylation should be strongly augmented upon expression of an MTS-lacking mutant protein in *cim3-1* cells. Indeed, multiple SUMOylated species of the import-incompetent Adh3 mutant variant (MTSΔ-Adh3^3HA^) strongly accumulated in proteasome mutants (Fig. S4, *E* and *F*) and MTS deletion enhanced the SUMOylation of Adh3 in both wild-type and *cim3-1* cells (Fig. 5*E*). We thus conclude that the SUMOylation of mitochondrial proteins is additively triggered upon import failure and inhibition of the proteasomal degradation system.

## Discussion

Our study identifies SUMO modification as an element of cellular protein quality control that acts on mitochondrial proteins. This adds to the growing list of experimental evidence that suggests functions of SUMO in cellular stress responses (1). In particular, we find that SUMOylation of mitochondrial proteins is induced upon failed mitochondrial import and when HSP70- or proteasome-dependent surveillance systems are defective. We therefore propose that SUMO serves as a mark for nonfunctional and nonimported, perhaps even import-incompetent, mitochondrial proteins.

Interestingly, SUMOylation is detectable on small pools of processed mitochondrial proteins even under normal growth conditions. As judged from the apparent cleavage of their N-terminal MTS, these proteins seem to initiate or to have initiated import. Currently it is unclear whether SUMO modification occurs before, concomitant with, or after the attempted import event. It can even be speculated, given that mitochondrial proteins are typically imported in an unfolded and extended conformation (59), that the attachment of a folded SUMO moiety to a precursor protein may stall the translocation.

In agreement with a function of SUMO in the quality control of cytosolic proteins, we also provide evidence that SUMOylation of mitochondrial proteins does not rely on mitochondrial import. Thus, SUMOylation appears to occur outside their native environment, where mislocalized proteins are potential sources of cellular stress. Indeed, recent reports have highlighted that accumulation of nonimported mitochondrial precursor proteins (termed mitochondrial precursor overaccumulation stress (mPOS)) is a challenge to cellular proteostasis (60, 61). These studies furthermore show that cells react to mitochondrial precursor overaccumulation stress with specific compensatory mechanisms such as a reduction of cytosolic translation and an increase in proteasome activity. We speculate that SUMOylation of mitochondrial proteins might be a further protective mechanism involved in the cytosolic proteostatic responses to mitochondrial stress (62).

Protein modification by SUMO has also been implicated in the quality control of other proteins including transcriptional regulators (63) and aggregation-prone proteins involved in neurodegenerative diseases (64, 65). It has been proposed that SUMO may act as a “solubility enhancer” (65), which reduces protein aggregation (66–71) and enables clearance of aggregates by the ubiquitin-proteasome system via recruitment of specific SUMO-binding factors (72). Notably, fostering physical interactions between SUMOylated proteins and binding partners that harbor so-called SUMO-interacting motifs (SIMs) is apparently one of the most prominent functions of SUMO (13, 73). It can therefore be speculated that SUMO modification will determine the fate of nonimported mitochondrial proteins by the recruitment of specific SUMO-interacting motif–containing interaction partners, which in turn could affect the targeting competence and solubility of a modified protein. Moreover, although SUMOylation is apparently not an essential requirement for the proteasomal degradation of nonimported mitochondrial proteins (Fig. S4, *C* and *D*), such factors might contribute to the clearance of specific protein pools and could target species that have failed to be degraded by the common ubiquitin-proteasome–dependent mechanism. At any rate, our data provide the first evidence for SUMO acting on aberrant and nonimported mitochondrial proteins.

Finally, we emphasize that SUMO modification, as we describe it here for mitochondrial substrates, will most likely not discriminate between mislocalized proteins based on their original destination. We therefore reason that the modification is unlikely to be specific for mitochondrial proteins and that nonimported proteins of other organelles could become modified in a similar fashion. Accordingly, it can be envisioned that such substrates will arise in particular upon organelle dysfunction, mistargeting, or stress-induced protein damage. Consistent with this idea, SUMOylation is strongly induced in the nucleus upon proteotoxic stress (3).

Studying the functions of SUMO in proteostasis is challenged by the multiple other ways through which SUMO regulates protein function. For proteins within their native environment it is therefore difficult to dissect quality control and other functions of SUMO. By contrast, SUMO modification of mitochondrial substrates appears to occur outside of their functional compartment. Thus, our study not only brings renewed attention to the multifaceted roles of SUMO as a component of the cellular proteostasis network but also opens up a new opportunity for revealing the functions of SUMO in protein quality control.

## Experimental procedures

### Yeast strains and plasmids

All yeast (*Saccharomyces cerevisiae*) strains and plasmids used in this study are listed in Tables S2 and S3, respectively.

### Identification of SUMO substrates by SILAC mass spectrometry

Enrichment of SUMO conjugates from yeast cells expressing ^His^SUMO followed by SILAC-based mass spectrometric analysis has been described previously (37, 38). In brief, yeast cells deficient in the biosynthesis of lysine and arginine (*lys1*Δ *arg4*Δ) were grown in synthetic complete (SC) media containing either unlabeled or heavy isotope–labeled lysine and arginine (Lys8, Arg10). ^His^SUMO conjugates were isolated using denaturing Ni-NTA pulldowns and separated on NuPAGE Bis-Tris Gels (4–12%) (Thermo Fisher Scientific). Gels were stained with Coomassie Blue and single lanes were excised in form of 10 separate gel slices. Subsequently, proteins were digested with trypsin or thermolysin and analyzed by LTQ Orbitrap mass spectrometry (74) and MaxQuant software (75).

SUMO-modified proteins were identified based on two criteria. The first was the enrichment in samples of yeast cells expressing ^His^SUMO compared with untagged SUMO (SILAC ratios above 2). The second was the detection of SUMO attachment sites as described previously (38).

We would like to note that the list of potential SUMO substrates presented here (Table S1) represents a compiled dataset from multiple mass spectrometry experiments.

### Yeast techniques and molecular cloning

Yeast deletion mutants and chromosomally tagged strains were generated by common PCR-based strategies, genetic crosses, and standard techniques (76, 77). Yeast strains were inoculated from fresh overnight cultures and grown using standard growth conditions (78). Typically, cells were cultured at 30 °C in yeast extract peptone dextrose (YPD) or SC media containing glucose (2%) as carbon source. For the expression of genes under the *GAL1* promoter, yeast cells (W303 background) were grown in medium containing 2% raffinose, and 2% galactose was added to induce protein expression. Strains harboring a temperature-sensitive *SSC1* allele (*ssc1-3*) were grown at 25 °C and shifted to 37 °C as indicated. The hypomorphic *SSA1* and *CIM3* mutants used in this study (*ssa1-45* and *cim3-1*) display phenotypes already at the permissive temperature. These strains including the corresponding wild-type controls were grown at 25 °C. For the qualitative analysis of growth phenotypes, exponentially growing yeast cultures were adjusted to an *A*_600_ of 1, and six 5-fold serial dilutions were spotted on SC agar plates. The plates were scanned after 2–3 days' incubation at 25 °C and 37 °C, respectively.

Plasmid constructs for the expression of ^His^SUMO under control of the *ADH1* promoter have been described previously (79, 80). Standard cloning techniques were used to generate constructs for the expression of HA-tagged proteins and N-terminally truncated mutant variants. Point mutations were introduced using PCR-based site-directed mutagenesis.

### Protein techniques, cellular fractionation, and Western blotting

Total cell extracts were prepared by TCA precipitation (76) and Ni-NTA pulldowns of ^His^SUMO conjugates under denaturing conditions were performed as described previously (37). SUMOylation of mitochondrial proteins in different yeast strains was initially analyzed with C-terminally 3HA-tagged proteins expressed from their endogenous promoters (Ilv6^3HA^ and Adh3^3HA^). To more quantitatively assess SUMOylation, expression systems under control of the *ADH1*, *GAL1,* or *TDH3* promoters were additionally used.

For ^His^SUMO Ni-NTA pulldowns combined with expression shut-off assays, cells were grown in YPD medium, shifted to 37 °C for 60 min, and treated with 0.5 mg/ml cycloheximide (CHX) dissolved in YPD medium prior to the experiment. Cells were harvested at the time points indicated in the respective experiment and denaturing Ni-NTA pulldowns were performed to isolate ^His^SUMO conjugates.

Cellular fractionations were performed as described previously (81) with minor modifications. Cells were lysed by bead-beating in lysis buffer (100 mm HEPES pH 7.5, 1% Triton X-100, 300 mm NaCl, EDTA-free protease inhibitor mixture (Roche), 1 mg/ml Pefabloc SC (Roche)) using zirconia/silica beads (Bio Spec Products, Inc., Bartlesville, OK) and a multi-tube bead-beater (MM301, Retsch Technology, Haan, Germany). Cellular lysates were cleared by centrifugation (2000 × *g*, 10 min, 4 °C) and the resulting total cell extracts (T fraction) were fractionated by centrifugation (16,000 × *g*, 10 min, 4 °C) to yield soluble (S fraction) and insoluble pellet (P fraction) fractions.

Proteins from cell extracts, cell fractions, or isolated by Ni-NTA pulldowns were separated on NuPAGE Bis-Tris gels (12% or 4–12%) (Thermo Fisher Scientific) and analyzed using standard Western blotting techniques.

### Cycloheximide shut-off assays

To monitor the degradation of import-incompetent Ilv6 (MTSΔ-Ilv6^3HA^) expressed from the *GAL1* promoter, cells were grown at 30 °C in SC medium containing 2% raffinose as carbon source. Protein expression was induced by addition of 2% galactose for 1 h and cells were shifted to 37 °C. Optionally, cells (*pdr5*Δ) were treated with 50 μm MG132 dissolved in DMSO. After 1 h at 37 °C cells were resuspended in YPD medium containing 0.5 mg/ml cycloheximide and samples of 1 *A*_600_ were taken at different time points. Cell extracts were prepared by TCA precipitation and analyzed by Western blotting.

### Fluorescence microscopy

Yeast cells (W303 background) were grown at 30 °C in SC medium containing 2% raffinose as carbon source. Cells were complemented with plasmids expressing full-length Ilv6^GFP^ from the endogenous promoter or import-incompetent Ilv6 (MTSΔ-Ilv6^GFP^) from the *GAL1* promoter. Protein expression was induced by addition of 2% galactose for 1 h. Cells were then transferred into a CellCarrier-96 black polystyrene microplate (PerkinElmer Life Sciences) and stained using Calcofluor White (Sigma-Aldrich). Images were captured at room temperature using an Opera Phenix HCS confocal microscope (PerkinElmer Life Sciences) equipped with an Olympus 63× water NA 1.15 objective.

### Antibodies

Polyclonal Smt3-specific antibodies were raised in rabbits and have been described previously (79). Monoclonal (F-7) and polyclonal (Y-11) antibodies directed against the HA epitope were purchased from Santa Cruz Biotechnology (Dallas, TX). The monoclonal Pgk1 (22C5D8) and Dpm1 (5C5) antibodies were from Thermo Fisher Scientific and the monoclonal HSP70 (BB70) antibody was from Enzo Life Sciences (Farmingdale, NY).

### Software

GraphPad Prism (GraphPad Software, La Jolla, CA) was used for data presentation (Fig. 1*A*) and ImageJ was used for Western blot quantification (Fig. 3, *B* and *D*). Microscopic imaging data were acquired and evaluated using Harmony 4.5 high-content imaging and analysis software (PerkinElmer Life Sciences).

## Author contributions

F.P. and S.J. conceived the study. F.P., F.d.B., I.P., B.P., and S.J. designed experiments and analyzed data. I.P. conducted and together with F.d.B. evaluated the initial mass spectrometric analysis. F.P. performed all other experiments. F.P., F.d.B., and B.P. wrote the manuscript.

## Supplementary Material

Supporting Information

## Footnotes

4 The abbreviations used are:

SUMO: small ubiquitin-like modifier
DRP1: dynamin-related protein 1
^His^SUMO: His-tagged SUMO
HSP70: heat shock protein 70
MTS: matrix-targeting sequence
Ni-NTA: nickel-nitrilotriacetic acid
SC: synthetic complete
SILAC: stable isotope labeling with amino acids in cell culture
YPD: yeast extract peptone dextrose.
