# Supplementary material for: Failed mitochondrial import and impaired proteostasis trigger SUMOylation of mitochondrial proteins
Source: J Biol Chem. 2017 Nov 28;293(2):599–609. doi: 10.1074/jbc.M117.817833 (PMC5767865; doi:10.1074/jbc.M117.817833)
Supplement: Supporting Information [file supp_293_2_599__index.html]

Failed mitochondrial import and impaired proteostasis trigger SUMOylation of mitochondrial proteins — Failed mitochondrial import and impaired proteostasis trigger SUMOylation of mitochondrial proteins — SUMOylation of mitochondrial proteins — Supporting Information 

# Failed mitochondrial import and impaired proteostasis trigger SUMOylation of mitochondrial proteins

## Supporting Information

- Supporting information (.pdf, 4.0 MB) - Supplemental figures S1-S4 and supplemental tables S2-S3
- Supplemental Table S1 (.xlsx, 37 KB) - Excel file containing a compiled list of potential mitochondrial SUMO substrates identified by multiple mass spectrometry experiments
