# Supplementary material for: Failed mitochondrial import and impaired proteostasis trigger SUMOylation of mitochondrial proteins
Source: J Biol Chem. 2017 Nov 28;293(2):599–609. doi: 10.1074/jbc.M117.817833 (PMC5767865; doi:10.1074/jbc.M117.817833)
Supplement: Supporting Information [file 10.1074_M117.817833_jbc.M117.817833-1.pdf]

Supplemental Data

Failed mitochondrial import and impaired proteostasis trigger SUMOylation of mitochondrial proteins

Florian Paasch, Fabian den Brave, Ivan Psakhye, Boris Pfander and Stefan Jentsch

Figure S1
Figure S2
Figure S3
Figure S4
Legend for Table S1 (provided as separate Excel file)
Table S2
Table S3
Supplemental references

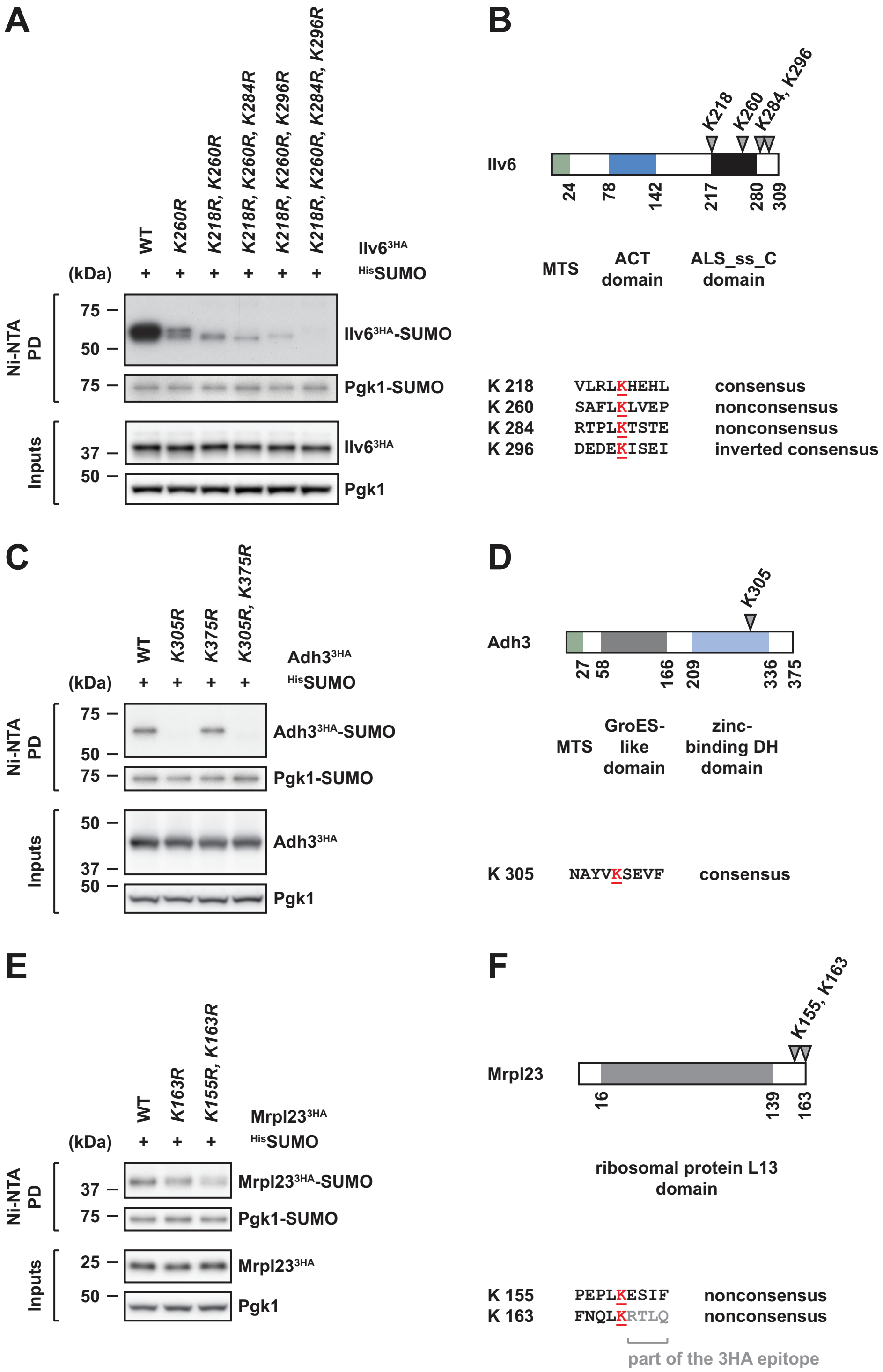

Figure S1. Mapping of SUMO acceptor sites of mitochondrial proteins.
(A, C, E) Identification of SUMO acceptor sites in Ilv6, Adh3 and Mrpl23. Denaturing ^{His}SUMO Ni-NTA pull-downs from cells harboring plasmids that express 3HA-tagged versions of Ilv6 (A), Adh3 (C) and Mrpl23 (E) or corresponding lysine-to-arginine mutants as indicated. Expression of 3HA-tagged proteins is under control of endogenous promoter (A), *GAL1* promoter (C) or *ADH1* promoter (E).

(B, D, F) Schematic representations of Ilv6 (B), Adh3 (D) and Mrpl23 (F) showing the positions of SUMO acceptor site lysines. Abbreviations: MTS (matrix-targeting sequence), ACT domain (named after aspartate kinase, chorismate mutase, TyrA), ALS_ss_C domain (acetolactate synthase, small subunit, C-terminal domain), zinc-binding DH domain (zinc-binding dehydrogenase domain).

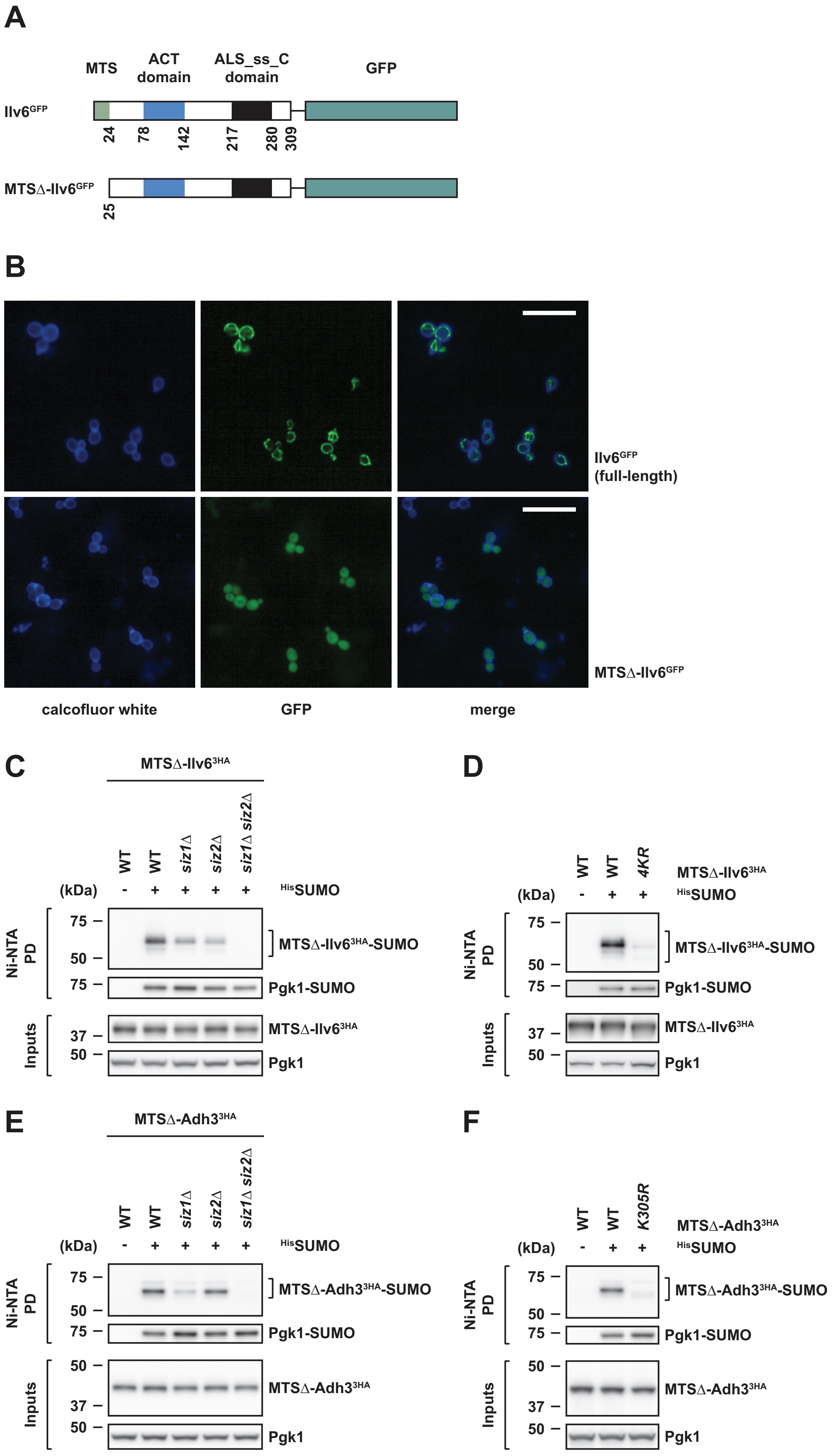
A
MTS
ACT domain
ALS_ss_C domain
GFP
Ilv6GFP
24
78
142
217
280
309
MTSΔ-Ilv6GFP
25
B
Ilv6GFP (full-length)
MTSΔ-Ilv6GFP
calcofluor white
GFP
merge
C
MTSΔ-Ilv63HA
WT
WT
siz1Δ
siz2Δ
siz1Δ siz2Δ
(kDa)
HisSUMO
Ni-NTA PD
MTSΔ-Ilv63HA-SUMO
Pgk1-SUMO
Inputs
MTSΔ-Ilv63HA
Pgk1
D
4KR
E
MTSΔ-Adh33HA
MTSΔ-Adh33HA-SUMO
F
K305R

Figure S2. SUMOylation of import-incompetent mutant variants of mitochondrial proteins.
(A, B) Generation and localization of an import-incompetent Ilv6 mutant variant. (A) Schematic representation of GFP fusion proteins of Ilv6 (Ilv6^{GFP}) and an import-incompetent variant lacking the N-terminal 24-amino-acid matrix-targeting sequence (MTSΔ-Ilv6^{GFP}). (B) Subcellular localization of wild type and import-incompetent versions of Ilv6^{GFP} using GFP fluorescence. Yeast cell walls were stained with calcofluor white. Scale bars: 20 µm.
(C-F) SUMOylation of Ilv6 and Adh3 variants lacking an MTS is mediated by the SUMO E3 ligases Siz1 and Siz2 and occurs at similar sets of SUMO acceptor sites as in wild type proteins. (C, E) Denaturing ^{His}SUMO Ni-NTA pull-downs from wild type (WT) cells and cells lacking the indicated SUMO E3 ligases. All strains harbor plasmids that express 3HA-tagged import-incompetent Ilv6 (MTSΔ-Ilv6^{3HA}) or Adh3 (MTSΔ-Adh3^{3HA}) from the *GAL1* promoter. (D, F) Denaturing ^{His}SUMO Ni-NTA pull-downs from cells expressing import-incompetent Ilv6 (MTSΔ-Ilv6^{3HA}) (D) or Adh3 (MTSΔ-Adh3^{3HA}) and corresponding lysine-to-arginine mutants as indicated from the *GAL1* promoter.

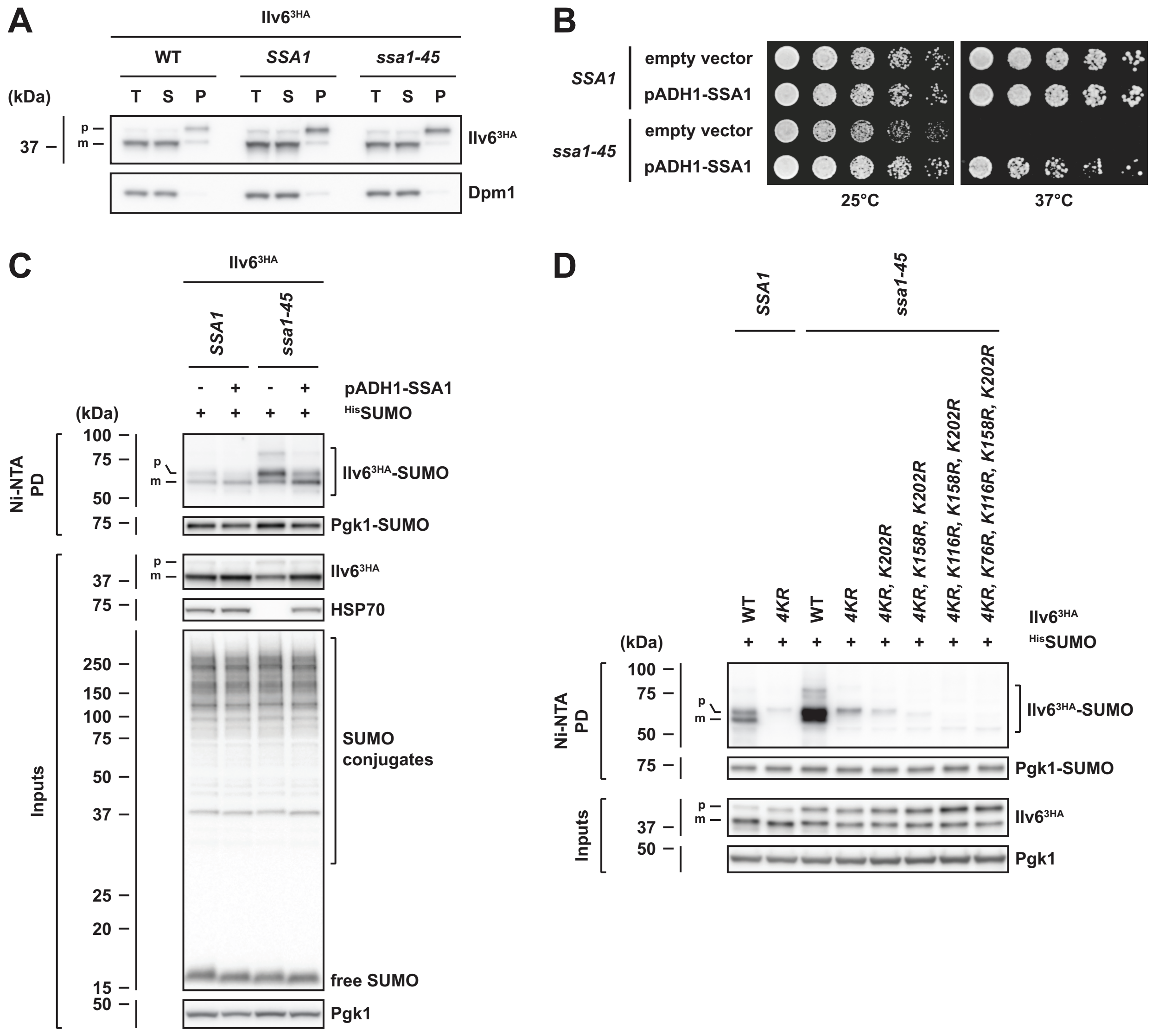

Figure S3. The SUMOylation pattern of Ilv6 is specifically altered in *SSA* mutant cells.

(A) Levels of the Ilv6 precursor are elevated in *SSA* mutant cells and show an increased propensity to fractionate as insoluble. Total (T) cell lysates of wild type (WT), *SSA1* and *ssa1-45* cells were separated into soluble (S) and insoluble pellet (P) fractions and analyzed by western blotting using HA epitope-specific, Dpm1-specific and Smt3-specific antibodies (fractions were loaded in a T:S:P ratio of 1:1:60). All strains express 3HA-tagged Ilv6 from the endogenous promoter and ^{His}SUMO from the *ADH1* promoter. Bands corresponding to the precursor protein (p) or mature form (m) are labeled.

(B, C) Ectopic expression of Ssa1 promotes growth of *ssa1-45* cells at restrictive temperature and reduces Ilv6 precursor SUMOylation in *SSA* mutants. (B) *SSA1* and *ssa1-45* cells were complemented with an empty vector or plasmids expressing wild type Ssa1 from the *ADH1* promoter. 5-fold serial dilutions of cells were spotted on SC agar plates and grown at 25°C for 2 days or 37°C for 3 days. (C) Denaturing ^{His}SUMO Ni-NTA pull-downs from *SSA1* and *ssa1-45* cells harboring plasmids that express wild type Ssa1 from the *ADH1* promoter. All strains express 3HA-tagged Ilv6 from the endogenous promoter. Bands corresponding to the unmodified or monoSUMOylated precursor protein (p) or mature form (m) are labeled.

(D) SUMOylation of multiple sites in the Ilv6 precursor is detectable in *SSA* mutant cells. Denaturing ^{His}SUMO Ni-NTA pull-downs from *SSA1* and *ssa1-45* cells harboring plasmids that express 3HA-tagged

wild type (WT) Ilv6 or one of multiple *KR* mutant variants from the *ADH1* promoter. Bands corresponding to the unmodified or monoSUMOylated precursor protein (p) or mature form (m) are labeled.

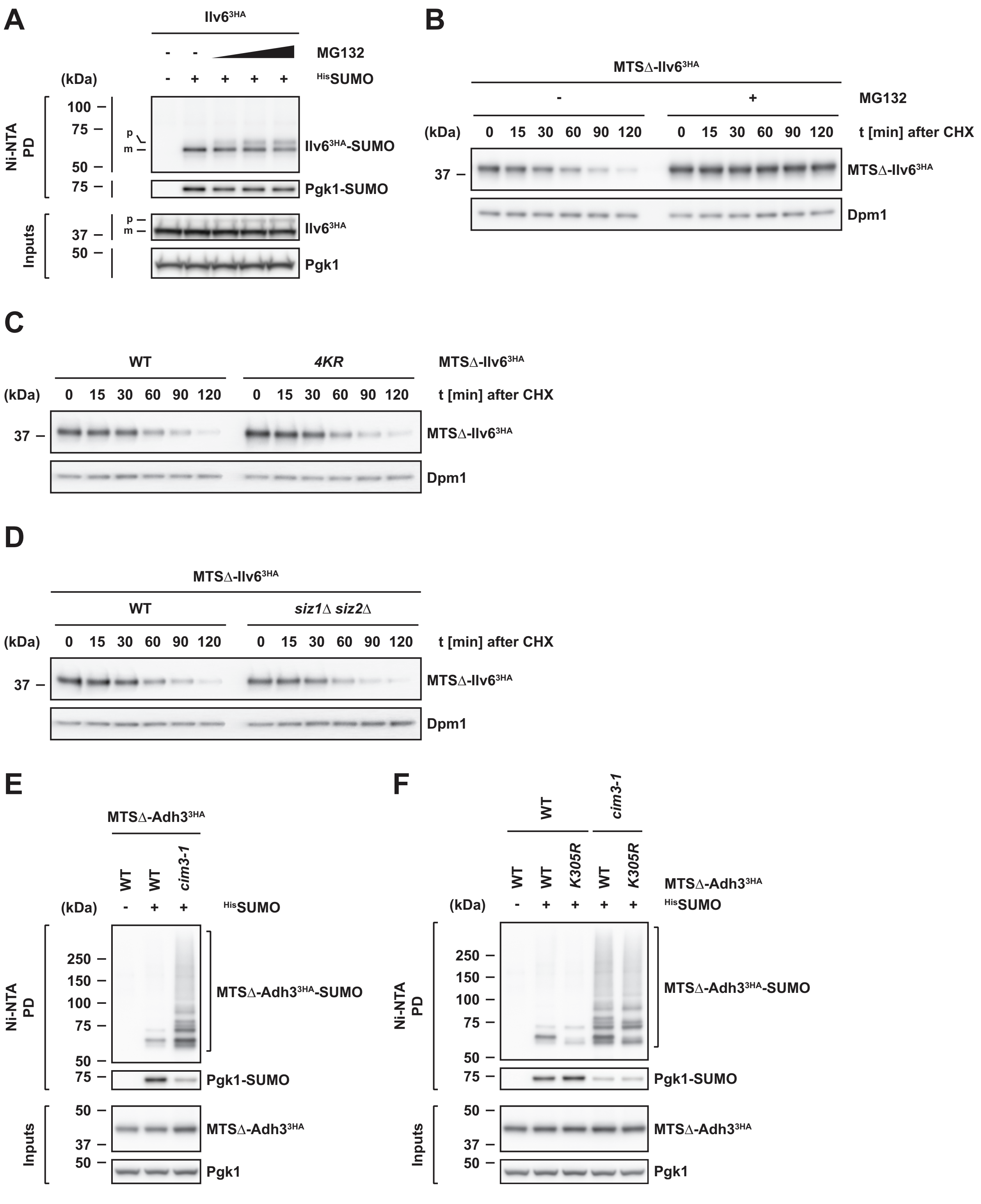

Figure S4. Enhanced SUMOylation of mitochondrial proteins in proteasome mutant cells.
(A) SUMOylation of the Ilv6 precursor is enhanced upon proteasome inhibition by MG132. Yeast cells (*pdr5Δ*) expressing C-terminally 3HA-tagged Ilv6 were treated with 10 µM, 20 µM or 50 µM MG132 for 2 h and denaturing Ni-NTA pull-downs were performed to isolate ^{His}SUMO conjugates. Bands

corresponding to the unmodified or monoSUMOylated precursor protein (p) or mature form (m) are labeled.
(B) Import-incompetent Ilv6 is degraded in a proteasome-dependent manner. Yeast cells (*pdr5Δ*) expressing import-incompetent Ilv6 (MTSΔ-Ilv6^{3HA}) from the *GAL1* promoter were incubated with MG132 for 1 h, treated with 0.5 mg/ml cycloheximide (CHX) and collected at the indicated time points. Mutant HA-tagged Ilv6 and Dpm1 were detected by western blotting using HA tag- and Dpm1-specific antibodies.
(C, D) Lack of Ilv6 SUMOylation does not noticeably affect the degradation of import-incompetent Ilv6. Experimental setup as in (B) but with cells expressing wild type (WT) or the *4KR* variant of import-incompetent Ilv6 (C) and wild type (WT) cells or cells lacking the SUMO E3 ligases Siz1 and Siz2 (*siz1Δ siz2Δ*) (D).
(E, F) SUMOylation of import-incompetent Adh3 is strongly enhanced in proteasome mutant cells. Denaturing ^{His}SUMO Ni-NTA pull-downs from wild type (WT) and *cim3-1* cells harboring plasmids that express MTSΔ-Adh3^{3HA} or the corresponding *K305R* variant from the *GAL1* promoter.

Table S1. Excel file containing a compiled list of potential mitochondrial SUMO substrates identified by multiple mass spectrometry experiments.

Table S2. Yeast (*Saccharomyces cerevisiae*) strains used in this study, related to Experimental procedures.

Strain	Genotype	Reference
DF5	*his3-Δ200 leu2-3,112 lys2-801 trp1-1 ura3-52*	(1,2)
W303 (*RAD5*)	*ade2-1 can1-100 his3-11,15 leu2-3,112 trp1-1 ura3-1 RAD5*	(3)
SSC1	*Matα, his4-713 lys2 ura3-52 trp1Δ leu2-3,112 SSC1*	(4)
ssc1-3	*Matα, his4-713 lys2 ura3-52 trp1Δ leu2-3,112 ssc1-3::LEU2*	(4)
JN516	*Matα, his3-11,15 leu2-3,112 his3-11 ura3-52 trp1Δ lys2 SSA1 ssa2Δ::LEU2 ssa3Δ::TRP1 ssa4Δ::LYS2*	(5)
ssa1-45 (ΔU)	*Matα, his3-11,15 leu2-3,112 his3-11 ura3-52 trp1Δ lys2 ssa1-45 ssa2Δ::LEU2 ssa3Δ::TRP1 ssa4Δ::LYS2*	(5,6)
CMY826	*Mata, ura3-52 leu2Δ1 his3Δ-200 trp1Δ63 lys2-801 ade2-101 bar1Δ::HIS3*	(7)
CMY726	*Matα, cim3-1 ura3-52 leu2Δ1*	(7)
Y0002	DF5, *Matα*	(2)
YFPX4-3A	DF5, *Matα, pADH-^{His}SMT3::URA3*	this study
YFPX10-5C	DF5, *Matα, ADH3^{3HA}::kanMX4*	this study
YFPX12-9B	DF5, *Matα, ILV6^{3HA}::kanMX4*	this study
YFPX14-1C	DF5, *Matα, pADH-^{His}SMT3::URA3 ADH3^{3HA}::kanMX4*	this study
YFPX16-6C	DF5, *Matα, pADH-^{His}SMT3::URA3 ILV6^{3HA}::kanMX4*	this study
YFPX244-5C	DF5, *Matα, pADH-^{His}SMT3::URA3 siz1Δ::hphNT1*	this study
YFPX245-7C	DF5, *Matα, pADH-^{His}SMT3::URA3 siz2Δ::natNT2*	this study
YFPX248-2C	DF5, *Matα, pADH-^{His}SMT3::URA3 siz1Δ::hphNT1 siz2Δ::natNT2*	this study
YFPX149-12D	DF5, *Matα, pADH-^{His}SMT3::URA3 ADH3^{3HA}::kanMX4 siz1Δ::hphNT1*	this study
YFPX147-4B	DF5, *Matα, pADH-^{His}SMT3::URA3 ADH3^{3HA}::kanMX4 siz2Δ::HIS3MX6*	this study
YFPX153-2D	DF5, *Matα, pADH-^{His}SMT3::URA3 ADH3^{3HA}::kanMX4 siz1Δ::hphNT1 siz2Δ::HIS3MX6*	this study
YFPX150-7C	DF5, *Matα, pADH-^{His}SMT3::URA3 ILV6^{3HA}::kanMX4 siz1Δ::hphNT1*	this study
YFPX164-3C	DF5, *Matα, pADH-^{His}SMT3::URA3 ILV6^{3HA}::kanMX4 siz2Δ::natNT2*	this study
YFPX165-14B	DF5, *Matα pADH-^{His}SMT3::URA3 ILV6^{3HA}::kanMX4 siz1Δ::hphNT1 siz2Δ::natNT2*	this study
Y2725	W303, *Matα*	(3)
YFP339	W303, *Matα, YIplac211-pADH-^{His}SMT3::URA3*	this study
YFP406	W303, *Matα, pdr5Δ::HIS3MX6*	this study
YFPX118-13C	W303, *Matα, siz1Δ::hphNT1 siz2Δ::HIS3MX6*	this study
YFP582	W303, *Matα, ILV6^{3HA}::TRP1 pdr5Δ::kanMX4*	this study
YFP522	W303, *Matα, YIplac211-pADH-^{His}SMT3::URA3 ILV6^{3HA}::TRP1 pdr5Δ::kanMX4*	this study
YFPX212-7D	W303, *Matα, YIplac211-pADH-^{His}SMT3::URA3 siz1Δ::hphNT1*	this study
YFPX213-7D	W303, *Matα, YIplac211-pADH-^{His}SMT3::URA3 siz2Δ::natNT2*	this study
YFPX220-20D	W303, *Matα, YIplac211-pADH-^{His}SMT3::URA3 siz1Δ::hphNT1 siz2Δ::natNT2*	this study
YFP627	W303, *Matα, pRS306-pGAL-ADH3^{3HA}-tCYC1::URA3*	this study
YFP630	W303, *Matα, pRS306-pGAL-adh3$_{28\text{-}375}^{3HA}$-tCYC1::URA3*	this study
YFPX251-15C	W303, *Matα, YIplac128-pADH-^{His}SMT3::LEU2 pRS306-pGAL-ADH3^{3HA}-tCYC1::URA3*	this study
YFPX256-9C	W303, *Matα, YIplac128-pADH-^{His}SMT3::LEU2 pRS306-pGAL-adh3$_{28\text{-}375}^{3HA}$-tCYC1::URA3*	this study
YFPX255-3B	W303, *Matα, YIplac128-pADH-^{His}SMT3::LEU2 pRS306-pGAL-adh3$_{28\text{-}375}^{3HA}$-K305R-tCYC1::URA3*	this study
YFPX254-6D	W303, *Matα, YIplac128-pADH-^{His}SMT3::LEU2 pRS306-pGAL-adh3$_{28\text{-}375}^{3HA}$-tCYC1::URA3 cim3-1*	this study
YFPX255-7D	W303, *Matα, YIplac128-pADH-^{His}SMT3::LEU2 pRS306-pGAL-adh3$_{28\text{-}375}^{3HA}$-K305R-tCYC1::URA3 cim3-1*	this study
YFPX271-19D	W303, *Matα, YIplac128-pADH-^{His}SMT3::LEU2 pRS306-pADH-ADH3^{3HA}-tCYC1::URA3*	this study
YFPX272-20A	W303, *Matα, YIplac128-pADH-^{His}SMT3::LEU2 pRS306-pADH-adh3$_{28\text{-}375}^{3HA}$-tCYC1::URA3*	this study
YFPX271-11D	W303, *Matα, YIplac128-pADH-^{His}SMT3::LEU2 pRS306-pADH-ADH3^{3HA}-tCYC1::URA3 cim3-1*	this study
YFPX272-19C	W303, *Matα, YIplac128-pADH-^{His}SMT3::LEU2 pRS306-pADH-adh3$_{28\text{-}375}^{3HA}$-tCYC1::URA3 cim3-1*	this study

(continued on next page)

YFP95	*Matα, SSC1 YIplac211-pADH-*^{His}*SMT3::URA3 ILV6*^{3HA}*::TRP1*	this study
YFP103	*Matα, ssc1-3 YIplac211-pADH-*^{His}*SMT3::URA3 ILV6*^{3HA}*::TRP1*	this study
YFP516	*Matα, SSA1 ssa2Δ::LEU2 ssa3Δ::TRP1 ssa4Δ::LYS2 YIplac211-pADH-*^{His}*SMT3::URA3*	this study
YFP519	*Matα, ssa1-45 ssa2Δ::LEU2 ssa3Δ::TRP1 ssa4Δ::LYS2 YIplac211-pADH-*^{His}*SMT3::URA3*	this study
YFP602	*Matα, SSA1 ssa2Δ::LEU2 ssa3Δ::TRP1 ssa4Δ::LYS2 YIplac211-pADH-*^{His}*SMT3::URA3 ADH3*^{3HA}*::kanMX4*	this study
YFP606	*Matα, SSA1 ssa2Δ::LEU2 ssa3Δ::TRP1 ssa4Δ::LYS YIplac211-pADH-*^{His}*SMT3::URA3 ILV6*^{3HA}*::kanMX4*	this study
YFP612	*Matα, ssa1-45 ssa2Δ::LEU2 ssa3Δ::TRP1 ssa4Δ::LYS2 YIplac211-pADH-*^{His}*SMT3::URA3 ADH3*^{3HA}*::kanMX4*	this study
YFP616	*Matα, ssa1-45 ssa2Δ::LEU2 ssa3Δ::TRP1 ssa4Δ::LYS2 YIplac211-pADH-*^{His}*SMT3::URA3 ILV6*^{3HA}*::kanMX4*	this study
YFP162	CMY826, *Mata, YIplac211-pADH-*^{His}*SMT3::URA3*	this study
YFP140	CMY763, *Matα, YIplac211-pADH-*^{His}*SMT3::URA3*	this study
YFP167	CMY826, *Mata, YIplac211-pADH-*^{His}*SMT3::URA3 ADH3*^{3HA}*::kanMX4*	this study
YFP154	CMY763, *Matα, YIplac211-pADH-*^{His}*SMT3::URA3 ADH3*^{3HA}*::kanMX4*	this study
YFP171	CMY826, *Mata, YIplac211-pADH-*^{His}*SMT3::URA3 ILV6*^{3HA}*::kanMX4*	this study
YFP156	CMY763, *Matα, YIplac211-pADH-*^{His}*SMT3::URA3 ILV6*^{3HA}*::kanMX4*	this study

Table S3. Plasmids used in this study, related to Experimental procedures.

Plasmid	Description	Reference
p41XADH	pRS41X-pADH-tCYC1	(8)
p41XGAL	pRS41X-pGAL-tCYC1	(9)
D1374	YIplac211-pADH-^{His}SMT3	(10)
D1549	YIplac128-pADH-^{His}SMT3	(11,12)
pFP17	YCplac22-pILV6-Ilv6^{3HA}	this study
pFP38	YCplac22-pILV6-Ilv6^{3HA}-K260R	this study
pFP41	YCplac22-pILV6-Ilv6^{3HA}-K218, K260R	this study
pFP50	YCplac22-pILV6-Ilv6^{3HA}-K218,K260R, K284R	this study
pFP51	YCplac22-pILV6-Ilv6^{3HA}-K218,K260R, K296R	this study
pFP52	YCplac22-pILV6-Ilv6^{3HA}-K218,K260R, K284R, K296R (4KR)	this study
pFP53	p415GAL-Ilv6^{3HA}	this study
pFP62	p415GAL-Ilv6$_{25\text{-}309}^{3HA}$	this study
pFP72	p415GAL-Ilv6$_{25\text{-}309}^{3HA}$-K218R, K260R, K284R, K296R (4KR)	this study
pFP108	p413ADH-Ilv6^{3HA}	this study
pFP109	p413ADH-Ilv6^{3HA}-K218R, K260R, K284R, K296R (4KR)	this study
pFP110	p413ADH-Ilv6^{3HA}-4KR, K202R	this study
pFP111	p413ADH-Ilv6^{3HA}-4KR, K158R, K202R	this study
pFP112	p413ADH-Ilv6^{3HA}-4KR, K116R, K158R, K202R	this study
pFP113	p413ADH-Ilv6^{3HA}-4KR, K76R, K116R, K158R, K202R	this study
pFP93	pRS313-pILV6-Ilv6^{GFP}	this study
pFP91	p413GAL-Ilv6$_{25\text{-}309}^{GFP}$	this study
pFP12	p415GAL-Adh3^{3HA}	this study
pFP23	p415GAL-Adh3^{3HA}-K305R	this study
pFP24	p415GAL-Adh3^{3HA}-K375R	this study
pFP25	p415GAL-Adh3^{3HA}-K305R, K375R	this study
pFP13	p415GAL-Adh3$_{28\text{-}375}^{3HA}$	this study
pFP107	p415GAL-Adh3$_{28\text{-}375}^{3HA}$-K305R	this study
pFP118	pRS413-pTDH3-Adh3^{3HA}	this study
pFP119	pRS413-pTDH3-Adh3^{3HA}-K305R	this study
pFP120	pRS413-pTDH3-Adh3$_{28\text{-}375}^{3HA}$	this study
pFP121	pRS413-pTDH3-Adh3$_{28\text{-}375}^{3HA}$-K305R	this study
pFP103	pRS306-pGAL-Adh3$_{28\text{-}375}^{3HA}$	this study
pFP106	pRS306-pGAL-Adh3$_{28\text{-}375}^{3HA}$-K305R	this study
pFP114	pRS306-pADH-Adh3^{3HA}	this study
pFP115	pRS306-pADH-Adh3$_{28\text{-}375}^{3HA}$	this study
pFP97	p415ADH-Mrpl23^{3HA}	this study
pFP98	p415ADH-Mrpl23^{3HA}-K163R	this study
pFP99	p415ADH-Mrpl23^{3HA}-K155R, K163R	this study
pFP77	p413ADH-Ssa1	this study

Plasmids encoding the import-incompetent mutant variant of Ilv6 (MTSΔ-Ilv6) encode a protein variant lacking the N-terminal 24 amino acids (Ilv6$_{25\text{-}309}$); plasmids encoding the import-incompetent mutant variant of Adh3 (MTSΔ-Adh3) encode a protein variant lacking the N-terminal 27 amino acids (Adh3$_{28\text{-}375}$).

Supplemental references

1. Finley, D., Ozkaynak, E., and Varshavsky, A. (1987) The yeast polyubiquitin gene is essential for resistance to high temperatures, starvation, and other stresses. *Cell* **48**, 1035-1046
2. Ulrich, H. D., and Jentsch, S. (2000) Two RING finger proteins mediate cooperation between ubiquitin-conjugating enzymes in DNA repair. *EMBO J* **19**, 3388-3397
3. McDonald, J. P., Levine, A. S., and Woodgate, R. (1997) The Saccharomyces cerevisiae RAD30 gene, a homologue of Escherichia coli dinB and umuC, is DNA damage inducible and functions in a novel error-free postreplication repair mechanism. *Genetics* **147**, 1557-1568
4. Gambill, B. D., Voos, W., Kang, P. J., Miao, B., Langer, T., Craig, E. A., and Pfanner, N. (1993) A dual role for mitochondrial heat shock protein 70 in membrane translocation of preproteins. *J Cell Biol* **123**, 109-117
5. Becker, J., Walter, W., Yan, W., and Craig, E. A. (1996) Functional interaction of cytosolic hsp70 and a DnaJ-related protein, Ydj1p, in protein translocation in vivo. *Mol Cell Biol* **16**, 4378-4386
6. Taxis, C., Hitt, R., Park, S. H., Deak, P. M., Kostova, Z., and Wolf, D. H. (2003) Use of modular substrates demonstrates mechanistic diversity and reveals differences in chaperone requirement of ERAD. *J Biol Chem* **278**, 35903-35913
7. Ghislain, M., Udvardy, A., and Mann, C. (1993) S. cerevisiae 26S protease mutants arrest cell division in G2/metaphase. *Nature* **366**, 358-362
8. Mumberg, D., Muller, R., and Funk, M. (1995) Yeast vectors for the controlled expression of heterologous proteins in different genetic backgrounds. *Gene* **156**, 119-122
9. Mumberg, D., Muller, R., and Funk, M. (1994) Regulatable promoters of Saccharomyces cerevisiae: comparison of transcriptional activity and their use for heterologous expression. *Nucleic Acids Res* **22**, 5767-5768
10. Hoege, C., Pfander, B., Moldovan, G. L., Pyrowolakis, G., and Jentsch, S. (2002) RAD6-dependent DNA repair is linked to modification of PCNA by ubiquitin and SUMO. *Nature* **419**, 135-141
11. Psakhye, I., and Jentsch, S. (2012) Protein group modification and synergy in the SUMO pathway as exemplified in DNA repair. *Cell* **151**, 807-820
12. Sacher, M., Pfander, B., Hoege, C., and Jentsch, S. (2006) Control of Rad52 recombination activity by double-strand break-induced SUMO modification. *Nat Cell Biol* **8**, 1284-1290
